# Predictors of Genetic Testing for Hereditary Cancer in Qualified Individuals

**DOI:** 10.18103/mra.v13i7.6661

**Published:** 2025-07

**Authors:** Liliana Arida-Moody, Sarah Austin, Erika N. Hanson, Emerson Delacroix, Erika Koeppe, John D. Rice, Matthew Demerath, Shayna Weiner, Jennifer J. Griggs, Kenneth Resnicow, Elena M. Stoffel

**Affiliations:** 1 Rogel Cancer Center, University of Michigan, Ann Arbor, Michigan, USA; 2 Department of Internal Medicine, University of Michigan School of Medicine, Ann Arbor, Michigan, USA; 3 University of Michigan School of Public Health, Ann Arbor, Michigan, USA

## Abstract

**Introduction and Methods:**

We surveyed individuals in the United States whose personal and/or family history met clinical criteria for genetic testing for cancer susceptibility. Using univariate and multivariable logistic regression analyses, we examined demographic and clinical factors associated with self-reported receipt of clinical genetic testing.

**Results:**

Of the 1,269 respondents with personal and/or family history meeting clinical criteria for genetic testing, 48.1% reported having undergone clinical genetic testing. Testing uptake varied by cancer type (p<0.05) and was highest among individuals with personal diagnoses of breast (73.9%), ovarian (88.2%), and pancreatic (70.2%) cancers, as compared with prostate cancer (33.6%). Females were significantly more likely than males to have undergone genetic testing (odds ratio, OR, 1.67, CI: 1.17–2.37), and people 70 years or older were less likely to have had testing when compared with those age under 50 (OR 0.33, CI: 0.22–0.48). Individuals who were diagnosed with cancer 15 or more years ago were less likely to have had genetic testing than those with more recent cancer diagnoses (OR 0.47 CI: 0.30–0.74). Individuals who were eligible to receive genetic testing based on their family history of cancer alone were less likely to have had testing than those with a personal cancer diagnosis (OR 0.65, CI: 0.44–0.97).

**Conclusion:**

While clinical genetic testing is routinely offered to specific groups of patients at risk for hereditary cancer syndromes (especially those with a personal or family history of breast and/or ovarian cancers), uptake remains low among men and among individuals who qualify for genetic testing based on diagnoses of non-gynecologic cancers and/or family history alone. Patient- and clinician-facing interventions are needed to raise awareness of indications for clinical genetic testing for cancer susceptibility.

## Introduction

Inherited predisposition is implicated in 1 in 10 advanced cancer diagnoses^1^. Two common, highly penetrant hereditary cancer syndromes are Lynch syndrome (pathogenic variants in *MLH1, MSH2/EPCAM, MSH6, PMS2*) and hereditary breast and ovarian cancer syndrome (HBOC, pathogenic variants in *BRCA1, BRCA2*) each of which affects roughly 1 in 300 individuals in the general population^2–5^. Clinical guidelines recommend genetic testing for cancer susceptibility for individuals with personal and/or family history of breast, ovarian, prostate, pancreatic, colorectal, and uterine cancer based on specific criteria, including age at diagnosis, pathology, cancer staging, and family history information^3,4^. Germline pathogenic variants are detected in 13%–24% of patients with these cancer types, and diagnosis of a hereditary cancer syndrome has implications for cancer treatment and prevention for patients and their families^6–9^.

Despite evidence supporting the benefits of germline genetic testing for cancer susceptibility, uptake of testing remains low. Even among patients diagnosed with high-risk breast cancer, only half of those eligible undergo genetic testing^10^. Uptake of genetic testing is even lower among patients with other cancer types. Although all individuals diagnosed with ovarian or pancreatic cancers qualify for genetic testing, this testing is only completed by 1 in 3 ovarian cancer patients and 1 in 5 pancreatic cancer patients^11,12^. Genetic testing uptake is even lower among individuals diagnosed with prostate or colorectal cancer at around 10%^3,13,14^. While individuals with these cancer types may be appropriate candidates for genetic testing and benefit from targeted therapy and heightened surveillance recommended after a genetic diagnosis, gaps in referrals and completing genetic testing exist.

Barriers to germline genetic testing exist at both the patient and provider level. Examples of patient factors include insurance concerns, both related to testing coverage and privacy of genetic information, limited knowledge about the usefulness of genetic information, emotional concerns, and logistical barriers (timing, access to genetic services)^14–16^. Provider barriers include suboptimal strategies for identifying high-risk individuals, limited knowledge of the genetic testing process, and limited genetics workforce, leading to delayed care and under-referring of eligible patients^14,15,17,18^.

There are disparities in uptake of genetic testing not only across cancer types, but also across sociodemographic groups. Based on statewide data collected by the Michigan Hereditary Cancer Network, over 80% of individuals undergoing genetic evaluation in the state of Michigan between 2016–2020 were non-Hispanic white and female^19^. Similarly, an analysis of four U.S. academic medical centers found rates of referral for genetic evaluation and testing for diagnoses of colorectal cancer were lower among Black and Hispanic patients than non-Hispanic white patients^13^.

The majority of previous research on uptake of genetic testing has centered on newly diagnosed cancer patients and focused primarily on HBOC-related cancers. The aim of this study was to examine correlates of genetic testing uptake among individuals who meet current genetic testing criteria for genetic susceptibility to cancer, including individuals with or without personal history of a cancer diagnosis.

## Methods

### STUDY DESIGN

This analysis examines data collected as part of a multi-phase U.S. NIH/NCI-funded project designed to improve identification of individuals with genetic susceptibility to cancer^20^. In the first phase of the study, individuals receiving care at academic and community healthcare settings throughout the state of Michigan were invited to complete the Family Health History Tool (FHHT), an electronic survey completed by patients that was designed to collect a comprehensive family history of cancer in biological relatives. Each respondent was asked to provide information about cancer diagnoses in first- and second-degree relatives (FDR and SDR, respectively), which the software then used to generate a 3-generation pedigree. Information about cancer types and ages at diagnosis in respondents and family members were used in clinical algorithms and genetic risk models (e.g. PREMM5) by the study team to identify individuals meeting clinical criteria for germline genetic testing for cancer susceptibility^21,22^.

Individuals meeting criteria for germline genetic testing based on FHHT responses were contacted by the study team by phone and email to ascertain whether or not they had previously completed genetic testing, as individuals who had not had genetic testing would be eligible to participate in a randomized trial testing interventions to promote uptake of germline genetic testing (NCT 05162846/NIH U01CA232827)^20^. Data for this analysis came from the FHHT participant responses and from the follow-up questions about genetic testing used to determine eligibility for the clinical trial.

### PARTICIPANT RECRUITMENT

A broad range of recruitment strategies were utilized to assemble this study population. Patients with upcoming medical appointments at participating oncology practices across the state of Michigan, and at gastroenterology and gastrointestinal endoscopy clinics affiliated with an academic medical center (University of Michigan) were sent email invitations to complete the FHHT. In addition, individuals who had previously received cancer treatment at the academic medical center were invited to complete the FHHT. Lastly, we undertook a public service campaign to “know your family history of cancer” using media (social, print, radio, and television) to direct potential participants from the public to self-register and receive a link to complete the FHHT using a QR code.

**Figure 1.**
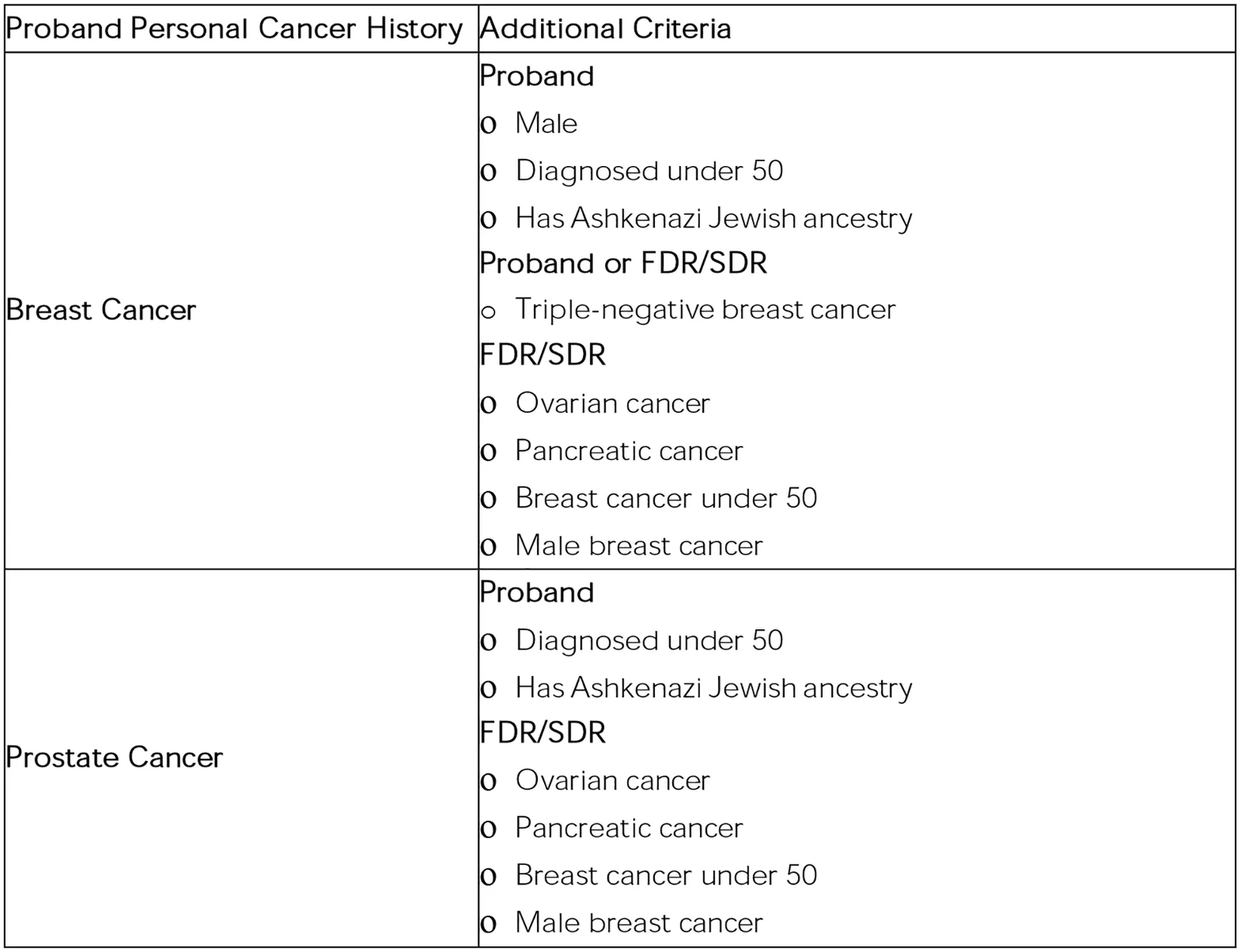

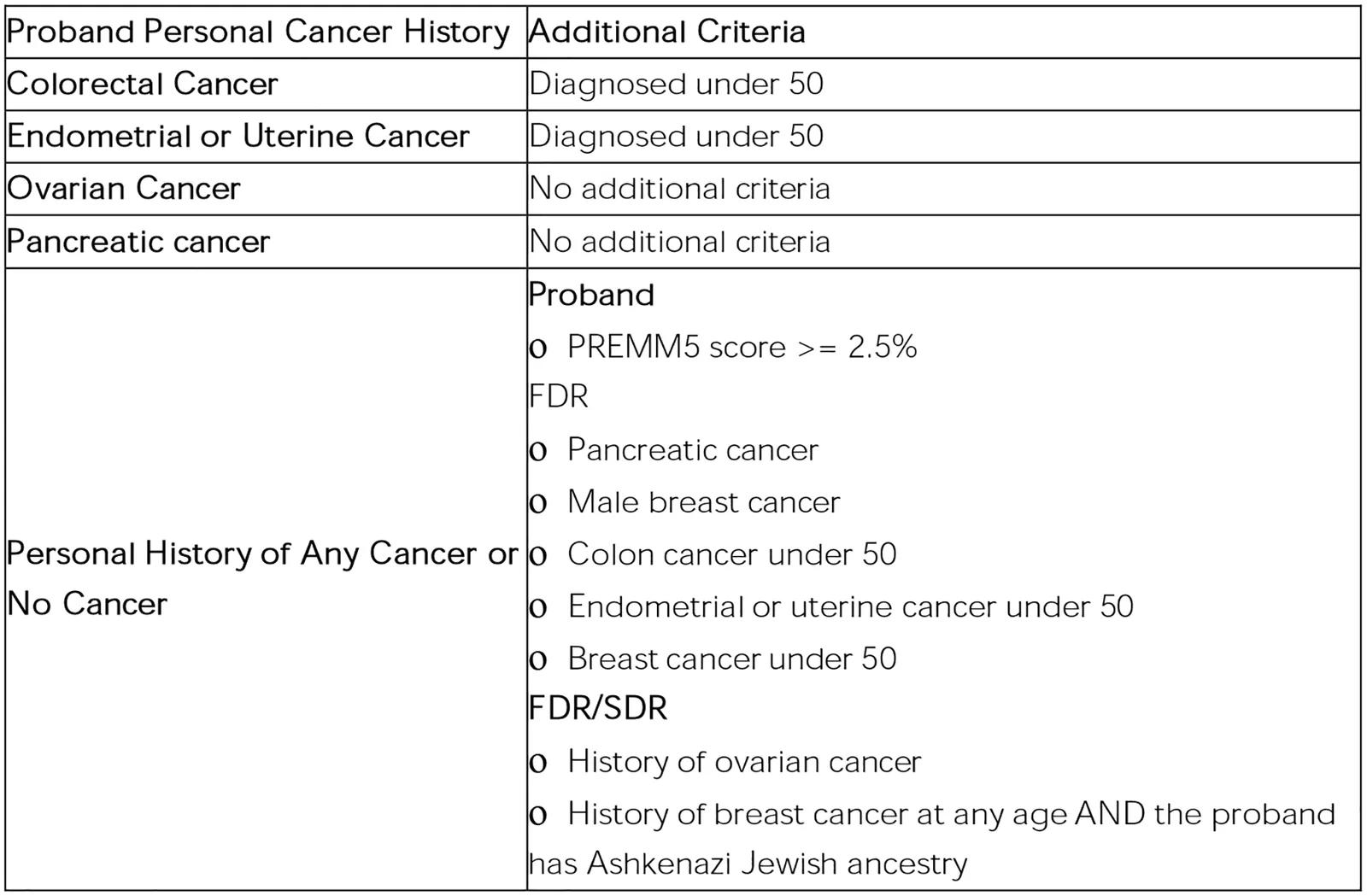
Clinical Criteria for Genetic Testing Eligibility To qualify for GT, proband must belong to one group in the left-hand column AND meet at least one of the corresponding criteria in the right- hand column. FDR, first degree relative; SDR, second degree relative.

All participants that completed the FHHT were given the opportunity to consent to be recontacted about a research study. Study team members reviewed participants’ self-reported personal and family histories of cancer and PREMM5 model scores to determine their eligibility for genetic testing based on a modified version of current clinical genetic testing guidelines (Figure 1)^3,4^. Individuals with no relevant personal history of cancer who met genetic testing criteria based only on a PREMM5 score between 2.5% and 5%, were reviewed for eligibility on a case-by-case basis by study team members with clinical genetics expertise.

Individuals who met clinical criteria for genetic testing and provided consent to be recontacted were invited to participate in a randomized clinical trial related to genetic testing (NCT 05162846/NIH U01CA232827). As part of the eligibility screening for the clinical trial, participants were asked by phone and email if they had ever undergone germline genetic testing for hereditary cancer. This information served as the primary outcome variable for these analyses.

This analysis includes data for the subset of individuals who completed the FHHT and for whom their genetic testing status could be ascertained (by participant self-report through completion of a survey sent through email or through telephone interview). We excluded participants that were deceased, those who reported having a genetic testing appointment scheduled, or those whose FHHT reports were identified as having errors in data entry. The University of Michigan Medical School Institutional Review Board (IRB) approved this study under HUM00192898.

Informed consent was obtained for those who had not previously had genetic testing. As this analysis includes data from all participants who were screened for the clinical trial, and screening data was collected prior to informed consent, the IRB approved a HIPAA waiver to include data from subjects (e.g. screen failures) who did not consent to the full trial.

### STATISTICAL ANALYSIS

We compared clinical characteristics of participants by testing status (previously completed genetic testing vs. no prior genetic testing). Univariate analyses consisted of Chi-square tests for categorical variables and t-tests for continuous variables. Demographic and clinical variables were also examined in a multivariable logistic regression model.

All continuous predictors were collapsed to facilitate interpretation of the logistic regression model. Demographic factors in this analysis included age, biological sex, race, ethnicity, and Ashkenazi Jewish ancestry. Clinical factors included personal cancer history (cancer types and ages at diagnosis). Forty-seven participants with missing or incomplete data for some variables were excluded from the logistic regression but are represented in the univariate analysis.

Although the 7 categories for proband race are shown in Table 1, we chose to dichotomize this variable (white vs. Other) in the regression due to small cell counts. Statistical analysis was performed using SPSS (Version 28). The deidentified datasets analyzed for this study are available from the corresponding author on reasonable request.

## Results

### STUDY SAMPLE

We invited 98,167 individuals to complete the FHHT. Of these 2,131 met clinical criteria for genetic testing and agreed to be recontacted by the study team. Of the 2,131 participants meeting criteria for genetic testing and agreeing to recontact, 1,322 provided response through phone or survey sent via email responses confirming their genetic testing status. We excluded 53 individuals who either had an appointment for genetic testing scheduled (n=39), were deceased (n=7), or had obvious data entry errors on their FHHT (n=7), leaving 1,269 individuals eligible for genetic testing that were included in analysis. In our study population, 1,007 (79.4%) participants had a personal history of cancer, 815 (64.2%) were female, 1,119 (88.2%) were white. Participants were an average 59.6 years of age (data not shown).

Among the 1,269 participants in this sample, 349 (27.5%) had been diagnosed with breast cancer, 101 (8.0%) with colorectal cancer, 57 (4.5%) with pancreatic cancer, 152 (12.0%) with prostate cancer, 59 (4.7%) with endometrial or uterine cancer, and 68 (5.4%) with ovarian cancer. A total of 262 participants (20.7%) had no personal history of cancer and qualified for genetic testing based on family history alone. Of all the participants eligible for genetic testing, 611 of 1,269 (48.2%) confirmed that they had previously undergone testing. Of the 815 female participants, 457 (56.1%) had undergone genetic testing whereas only 154 of the 454 male participants (33.9%) had undergone testing. With regard to age, 44.1% of participants over the age of 50 at enrollment reported testing compared to 60.8% of those under 50 (Table 1).

Testing rates differed among cancer types with rates of genetic testing highest among individuals with a personal history of triple negative breast cancer (TNBC, 95.2%). Of the remaining participants with breast cancer (non-TNBC) who met criteria for genetic testing, 71.0% had completed genetic testing. Among participants with colorectal cancer, 58.4% had completed genetic testing, whereas 70.2% of patients with pancreatic cancer and 88.2% of participants with ovarian cancer reported having completed genetic testing (Table 2). Participants in this sample with a personal diagnosis of cancer qualifying for HBOC criteria reported being tested at a higher rate (65.3%) than those diagnosed with Lynch syndrome-associated cancers (51.3%).

Among individuals with no personal history of cancer who qualified for genetic testing based on their family history alone, only 36.3% reported having completed testing.

In a multivariable logistic regression model that adjusted for demographic and clinical factors, female participants were significantly more likely than their male counterparts to have undergone genetic testing (OR 1.67, CI 1.17–2.37). Compared to individuals under the age of 50, those aged 50–69 were less likely to undergo testing (OR 0.53, CI 0.38–0.73), as were those 70 or older (OR 0.33, CI 0.22–0.48).

Compared to individuals with any cancer diagnosis (ever), those with no personal history of cancer (meeting genetic testing criteria based on family history alone) did not report significantly different rates of genetic testing (OR 0.90, CI 0.62–1.30, data not shown). However, when compared to the subset of individuals with a recent cancer diagnosis (within the last 4 years), participants without a personal cancer history were less likely to have completed testing (OR 0.65, CI 0.44–0.97).

A diagnosis of breast cancer was strongly associated with completion of genetic testing. Individuals with history of breast cancer (non-TNBC) had a four-fold increase in the odds of having had genetic testing compared to individuals without a breast cancer diagnosis (OR 4.33, CI 2.89–6.49), and those with a TNBC were had over a 30-fold increase in the odds of being tested compared with individuals without a breast cancer diagnosis (OR 32.8, CI 7.54–142.44). Individuals with a personal diagnosis of colorectal, pancreatic, or ovarian cancer meeting criteria for genetic testing were also more likely to have undergone genetic testing compared with those without a cancer diagnosis (colorectal: OR 1.95, CI 1.20–3.17; pancreatic: OR 5.46, CI 2.82–10.59; ovarian: OR 6.23, CI 2.66–14.60) (Table 3).

Time since the most recent cancer diagnosis was a significant predictor of completion of genetic testing in both univariate (data not shown) and multivariate adjusted analyses. Individuals who were diagnosed with their cancers 15 or more years prior to this study were significantly less likely to have undergone genetic testing when compared with individuals with more recent cancer diagnoses between 0 and 4 years prior to this study (OR 0.47, CI 0.30–0.74) (Table 3).

## Discussion

Our data demonstrate gaps in genetic testing uptake among individuals who meet clinical criteria for evaluation for hereditary cancer syndromes. Despite all participants meeting genetic testing criteria, only 48.1% of eligible individuals reported completing genetic testing, with testing rates highest among individuals with a personal history of breast cancer. In adjusted analyses, women were more likely than men to have undergone genetic testing, older individuals were less likely to have undergone testing than their younger counterparts, and individuals whose cancers were diagnosed more than 15 years ago were less likely to complete testing than those with more recent diagnoses.

Our finding that fewer than half of individuals eligible for genetic testing in this cohort had undergone testing is notable, albeit difficult to compare to prior published studies which have focused on individuals with specific cancer diagnoses or personal history of cancer^23–27^. A 2018 study by Barcenas et al. found that 24.1% of patients with triple-negative breast cancer had genetic testing compared to 95% in our cohort, which suggests that genetic testing is being better integrated in oncology care for breast cancer, especially for high-risk subtypes^28^. However, the disparity in genetic testing rates among those diagnosed with different cancer types and between those meeting clinical criteria for HBOC syndrome and Lynch syndrome in this sample raises concerns that the improvements in gene testing clinical integration beyond breast cancer is lagging. Our findings are consistent with recent studies that have shown that patients at risk for Lynch syndrome are under recognized, and that Lynch syndrome remains under-diagnosed compared to HBOC syndrome – despite having similar prevalence rates, affecting approximately 1 in 300 in the general population^2,27,29–31^.

Our finding that women are more likely than men to have had genetic testing is well-documented and may be attributed in part to increased awareness among oncologists, gynecologists, and patients of the need for genetic testing for diagnoses of breast and ovarian cancer^32,33^.

Differences in attitudes and motivations towards getting genetic testing may also play a role in this gap. Our finding that individuals without a personal history of cancer or with less recent cancer diagnoses are less likely to have undergone genetic testing is consistent with prior studies and highlights both the impact of recently-expanded clinical criteria for genetic testing and the need to implement strategies to identify at-risk individuals who may benefit from clinical genetic testing who receive their care outside of oncology settings^24, 27^. Genetic risk assessment is particularly important for this population, as they can pursue increased cancer screening and cancer prevention strategies prior to ever developing a cancer.

While ours is one of the largest studies to include participants meeting criteria for genetic testing based on personal and/or family history of multiple different cancer types, we recognize several limitations. The modified genetic testing criteria we employed for identifying individuals eligible for genetic testing (Figure 1) was not comprehensive and did not include criteria for gastrointestinal polyposis and other less common hereditary cancer syndromes; thus our strategy likely failed to identify some individuals for whom genetic testing would be clinically indicated. In addition, the information used to identify eligibility for genetic testing and genetic testing status was collected via patient self-report, which may not be entirely accurate. We were able to confirm that at least 5 participants who reported they had not undergone genetic testing later discovered they had previously completed genetic testing after discussing further with their physician. We limited our cohort to include only individuals who could confirm whether they had or had not completed genetic testing; however we had to exclude 40% of individuals because we were unable to ascertain their genetic testing status. Therefore, it is possible that our finding that half of eligible individuals have had genetic testing may reflect response bias, possibly overestimating rates of uptake of genetic testing.

Racial and ethnic disparities in genetic testing access and uptake are well-documented^24,33 34^. In this sample, recruited largely from a single U.S. academic medical center, the proportions of participants identifying as Black, Asian, Middle Eastern or North African, Native American, and non-white LatinX were each less than 3%, limiting our power for comparing rates of genetic testing uptake by race-ethnicity. Further study is needed to evaluate the generalizability of these findings for more diverse populations.

This cross-sectional analysis reflects genetic testing status at a single point in time. More meaningful causal relationships about barriers and motivators for genetic testing could be better inferred through longitudinal data, which we hope to address as part of the associated clinical trial of interventions promoting uptake of genetic testing^20^.

Despite these limitations, our work has implications for measurable improvement of genetic testing uptake. Additional education regarding genetic testing for Lynch syndrome and other cancer syndromes in addition to HBOC is needed for both patients and clinicians. The success of the campaign for patient and clinician awareness of *BRCA1/BRCA2* variants and their role in breast and ovarian cancer pathogenesis can be a model for how to better reach populations at risk for Lynch syndrome^35^. Further research is warranted on the reasons for observing lower testing rates among older patients as it has been shown that older individuals tend to have similar attitudes towards genetic testing as younger people^36^. It is possible that older cancer patients face additional barriers to genetic testing that may be addressed through provider-level or system- level interventions or may have different perceptions of their cancer risk and/or the usefulness of genetic testing.

Clinical criteria for who should undergo genetic testing for cancer susceptibility have expanded during the past two decades, and cancer survivors who may not have qualified for genetic testing at the time of their initial diagnosis may now be eligible for clinical testing. Genetic testing can still prove clinically useful many years after a cancer diagnosis, impacting cancer screening recommendations for the cancer survivor as well as their relatives. Furthermore, many individuals who have never been diagnosed with cancer qualify for germline genetic testing due to their family history of cancer. Identifying individuals at increased risk for cancer without a personal history of cancer (or with a remote cancer diagnosis) requires that clinicians in primary care and other non-oncology settings become familiar with criteria for genetic testing for cancer susceptibility and ascertain a complete family history.

## Conclusion

Genetic testing uptake differed by cancer type, sex, age, and recency of cancer diagnosis in our study showing there are gaps in uptake of hereditary cancer genetic testing. While genetic testing was completed for the majority of patients with breast and ovarian cancer, uptake remains low for individuals who qualify for genetic testing based on diagnoses of non-gynecologic cancers and/or family history alone. Additional interventions to overcome patient barriers to genetic testing, improve clinician knowledge on rapidly evolving genetic testing guidelines, and streamline the genetic testing process are needed. Integrating a systematic approach to collecting a comprehensive family cancer history and re-assessing cancer risks at multiple timepoints is a critical first step to improve identification of individuals that would benefit from genetic testing and specialized cancer screening and surveillance.

## Figures and Tables

**Table 1.** Demographic Characteristics by Genetic Testing Status (N=1,269)

		Did not have genetic testing (n=658)	Had genetic testing (n=611)	P-values
Age at enrollment (Mean + SD)		61.6 ± 14.3	57.4 ± 13.1	<0.001
Female		358 (54.4%)	457 (74.8%)	<0.001
Race	African American	10 (1.5%)	14 (2.3%)	0.16
	Asian	14 (2.1%)	11 (1.8%)	
	Middle Eastern North African	8 (1.2%)	8 (1.3%)	
	Multiple	12 (1.8%)	20 (3.3%)	
	Native American	7 (1.1%)	1 (0.2%)	
	Other/Unknown	27 (4.1%)	18 (2.9%)	
	White	580 (88.2%)	539 (88.2%)	
Ethnicity	LatinX	15 (2.3%)	9 (1.5%)	0.30
	Not LatinX	636 (96.7%)	589 (96.4%)	
	Unknown	7 (1.1%)	13 (2.1%)	
Ashkenazi Jewish Ancestry		107 (16.3%)	73 (12.0%)	0.03
Personal History of Cancer		491 (74.6%)	516 (84.5%)	<0.001

**Table 2.** Proband Cancer Diagnoses by Genetic Testing Status

		Did not have genetic testing (n=658)	Had genetic testing (n=611)	P-values
Breast Cancer	Non-TNBC	89 (29.0%)	218 (71.0%)	<0.001
	TNBC	2 (4.8%)	40 (95.2%)	
Colorectal Cancer		42 (41.6%)	59 (58.4%)	0.03
Pancreatic Cancer		17 (29.8%)	40 (70.2%)	<0.001
Prostate Cancer		101 (66.4%)	51 (33.6%)	<0.001
Endometrial or Uterine Cancer		36 (61.0%)	23 (39.0%)	0.15
Ovarian Cancer		8 (11.8%)	60 (88.2%)	<0.001

TNBC = triple negative breast cancer

**Table 3.** Logistic Regression Model for Completion of Genetic Testing (n=1,222)

	Odds Ratio	95% CI for Odds Ratio	
		Lower	Upper
Age (years) (ref: < 50)	–	–	–
**50 – 69**	**0.53**	**0.38**	**0.73**
**70+**	**0.33**	**0.22**	**0.48**
**Female (ref: male)**	**1.67**	**1.17**	**2.37**
Non-white Race (ref: White)	0.82	0.54	1.27
LatinX Ethnicity	0.78	0.30	2.07
Ashkenazi Jewish Ancestry	0.86	0.59	1.24
Breast Cancer (ref: no diagnosis)	–	–	–
**Non-TNBC Diagnosis**	**4.33**	**2.89**	**6.49**
**TNBC Diagnosis**	**32.77**	**7.54**	**142.44**
**Colorectal Cancer**	**1.95**	**1.20**	**3.17**
**Pancreatic Cancer**	**5.46**	**2.82**	**10.59**
Prostate Cancer	1.50	0.93	2.41
Endometrial or Uterine Cancer	0.64	0.34	1.21
**Ovarian Cancer**	**6.23**	**2.66**	**14.60**
Recency of Last Cancer Diagnosis (ref: 0–4 years)	–	–	–
5 – 14 years	0.80	0.57	1.13
**15+ years**	**0.47**	**0.30**	**0.74**
**No Cancer/Family History Only**	**0.65**	**0.44**	**0.97**

Significant values bolded, TNBC = triple negative breast cancer
